# Supplementary material for: Causal Relationship between Aging and Anorexia Nervosa: A White-Matter-Microstructure-Mediated Mendelian Randomization Analysis
Source: Biomedicines. 2024 Aug 16;12(8):1874. doi: 10.3390/biomedicines12081874 (PMC11351342; doi:10.3390/biomedicines12081874)
Supplement: Supplementary file 1 [file biomedicines-12-01874-s001.zip › Supplementary Figure/Figure S9/Leaveoneout plot of MR analysis for ubm-b-1819 to AN.pdf]

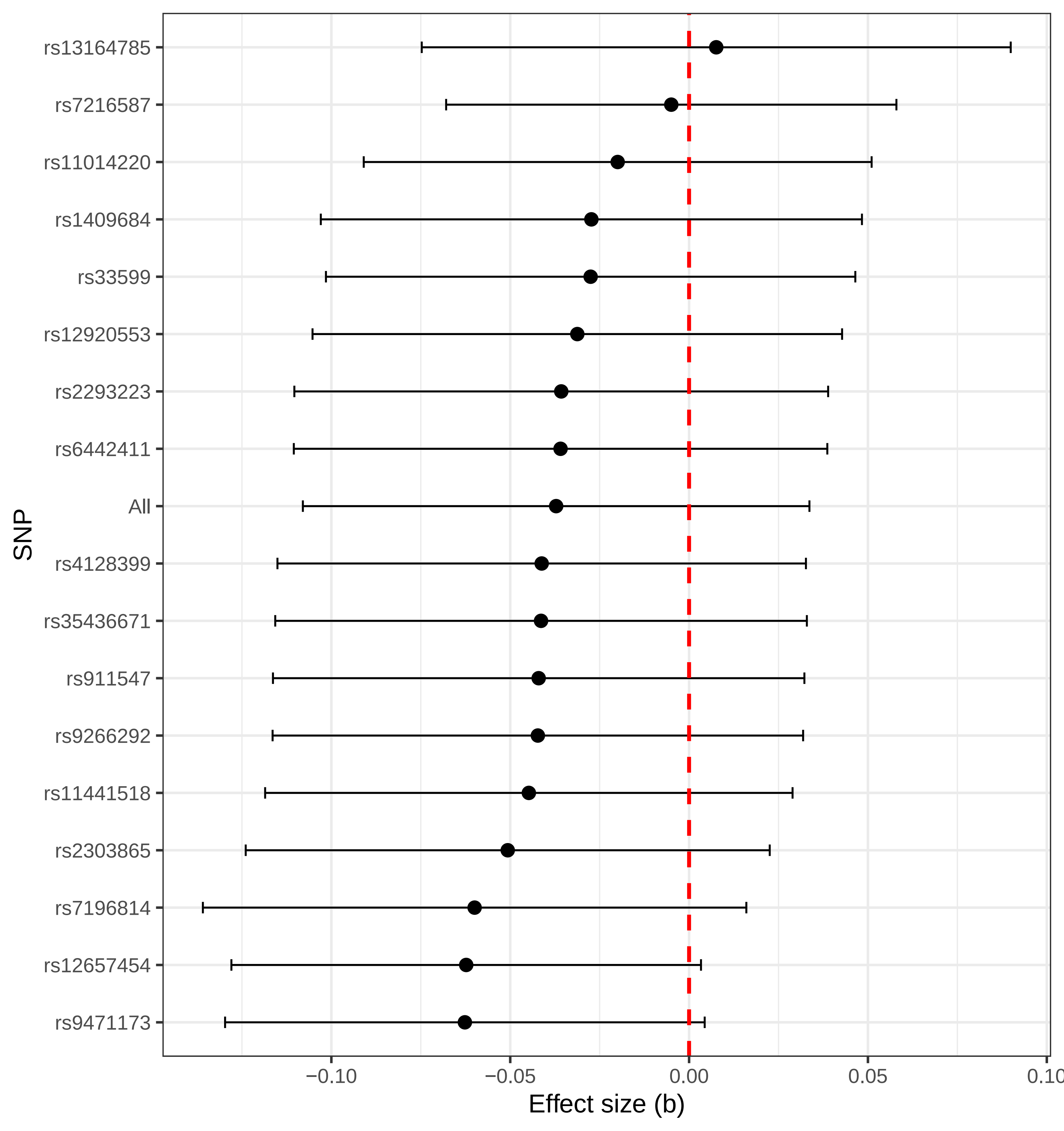

rs13164785
rs7216587
rs11014220
rs1409684
rs33599
rs12920553
rs2293223
rs6442411
All
rs4128399
rs35436671
rs911547
rs9266292
rs11441518
rs2303865
rs7196814
rs12657454
rs9471173
SNP
−0.10
−0.05
0.00
0.05
0.10
Effect size (b)
